# Development of Molecular Markers Linked to Powdery Mildew Resistance Gene *Pm4b* by Combining SNP Discovery from Transcriptome Sequencing Data with Bulked Segregant Analysis (BSR-Seq) in Wheat

**DOI:** 10.3389/fpls.2018.00095

**Published:** 2018-02-14

**Authors:** Peipei Wu, Jingzhong Xie, Jinghuang Hu, Dan Qiu, Zhiyong Liu, Jingting Li, Miaomiao Li, Hongjun Zhang, Li Yang, Hongwei Liu, Yang Zhou, Zhongjun Zhang, Hongjie Li

**Affiliations:** ^1^The National Key Facility for Crop Gene Resources and Genetic Improvement, Institute of Crop Sciences, Chinese Academy of Agricultural Sciences, Beijing, China; ^2^Department of Plant Pathology, China Agricultural University, Beijing, China; ^3^Institute of Genetics and Developmental Biology, Chinese Academy of Sciences, Beijing, China; ^4^College of Chemistry and Environment Engineering, Pingdingshan University, Pingdingshan, China

**Keywords:** powdery mildew, *Pm4b*, BSR-Seq, RNA-Seq, SNP, SSR marker

## Abstract

Powdery mildew resistance gene *Pm4b*, originating from *Triticum persicum*, is effective against the prevalent *Blumeria graminis* f. sp. *tritici* (*Bgt*) isolates from certain regions of wheat production in China. The lack of tightly linked molecular markers with the target gene prevents the precise identification of *Pm4b* during the application of molecular marker-assisted selection (MAS). The strategy that combines the RNA-Seq technique and the bulked segregant analysis (BSR-Seq) was applied in an F_2:3_ mapping population (237 families) derived from a pair of isogenic lines VPM1/7^∗^Bainong 3217 F_4_ (carrying *Pm4b*) and Bainong 3217 to develop more closely linked molecular markers. RNA-Seq analysis of the two phenotypically contrasting RNA bulks prepared from the representative F_2:3_ families generated 20,745,939 and 25,867,480 high-quality read pairs, and 82.8 and 80.2% of them were uniquely mapped to the wheat whole genome draft assembly for the resistant and susceptible RNA bulks, respectively. Variant calling identified 283,866 raw single nucleotide polymorphisms (SNPs) and InDels between the two bulks. The SNPs that were closely associated with the powdery mildew resistance were concentrated on chromosome 2AL. Among the 84 variants that were potentially associated with the disease resistance trait, 46 variants were enriched in an about 25 Mb region at the distal end of chromosome arm 2AL. Four *Pm4b*-linked SNP markers were developed from these variants. Based on the sequences of Chinese Spring where these polymorphic SNPs were located, 98 SSR primer pairs were designed to develop distal markers flanking the *Pm4b* gene. Three SSR markers, *Xics13*, *Xics43*, and *Xics76*, were incorporated in the new genetic linkage map, which located *Pm4b* in a 3.0 cM genetic interval spanning a 6.7 Mb physical genomic region. This region had a collinear relationship with *Brachypodium distachyon* chromosome 5, rice chromosome 4, and sorghum chromosome 6. Seven genes associated with disease resistance were predicted in this collinear genomic region, which included C2 domain protein, peroxidase activity protein, protein kinases of PKc_like super family, Mlo family protein, and catalytic domain of the serine/threonine kinases (STKc_IRAK like super family). The markers developed in the present study facilitate identification of *Pm4b* during its MAS practice.

## Introduction

In wheat (*Triticum aestivum* L.), powdery mildew is caused by the biotrophic fungus *Blumeria graminis* f. sp. *tritici* (*Bgt*) (Green et al., 2014). The epidemics of powdery mildew often occur in the wheat producing regions with cool and humid climates (Cowger et al., 2012). In China, this foliar disease is endangering most regions of winter wheat and spring wheat productions. In the last decade, the proper management measures can retrieve about 1.4984 million metric tons of yield losses, and the actual annual grain loss caused by powdery mildew was limited to 0.3045 million metric tons (Liu et al., 2016).

The use of host resistance is a commonly recognized means to reduce the economic losses and to control the epidemics of diseases (Hulbert et al., 2001). The development of powdery mildew-resistant wheat cultivars requires the availability of resistance genes. At present, 78 permanently designated and many other temporally designated powdery mildew resistance genes or alleles have been documented^1^. Some of them have single alleles, while others have multiple alleles (e.g., *Pm1*, *Pm2*, *Pm3*, *Pm4*, *Pm5*, and *Pm24* loci) (Hsam et al., 1998; Singrün et al., 2003; Hao et al., 2008, 2015; Bhullar et al., 2009, 2010; Xie et al., 2012; Zhang et al., 2016). The French cultivar VPM1 resistant to powdery mildew was developed from a complicated interspecific cross involving *Aegilops ventricosa* (Zhuk.) Chennav, and *T. persicum* Vav. (syn. *T. turgidum* var. *carthlicum* Nevski.) (Doussinault et al., 1983). A powdery mildew resistance gene in VPM1 was localized on a *T. persicum* chromosomal segment that was translocated onto the long arm of wheat chromosome 2A, and proved to be an allele in locus *Pm4*, designated *Pm4b* (Bariana and McIntosh, 1994). Even though it was identified over 30 years ago, *Pm4b* is still effective in certain regions of China and the United States (Wang et al., 2005; Parks et al., 2008; Zeng et al., 2014). It was also used to enhance powdery mildew resistance in triticale (× *Triticosecale* Wittmack) (Kowalczyk et al., 2011).

Great efforts have been taken to tag *Pm4b* and other *Pm4* alleles. Due to the alien origin nature and low abundance of the markers used, e.g., restriction fragment length polymorphism (RFLP) and its conversion of sequence-tagged site (STS), random amplified polymorphic DNA (RAPD), simple sequence repeats (SSRs), and sequence-related amplified polymorphism (SRAP), previous works on the molecular mapping of *Pm4b* did not generate closely linked molecular markers. Based on the RFLP marker *BCD1231* linked to *Pm4a*, Chen et al. (2002) designed an STS marker *STS*_470_ and mapped it 3.0 cM away from *Pm4b*. Another STS marker *STS*_-241_ developed from a cloned RAPD fragment was 4.9 cM from *Pm4b* (Yi et al., 2008). In that work, the SRAP marker *Me8*/*Em7*_-220_ was mapped 7.1 cM away from *Pm4b*. In an attempt to map *Pm4c*, Hao et al. (2008) also tested the *Pm4*-linked markers using a mapping population derived from the cross between VPM1 and the susceptible wheat Chancellor. An SSR marker *Xbarc122* (2.0 cM from the target gene) was closer to *Pm4b* than other existing molecular markers. Using the near-isogenic line (NIL) CI 14124, Ma et al. (2004) mapped *Pm4a* by screening 46 pairs of microsatellite primers, and determined that the genetic distance between SSR marker *Xgwm356* and *Pm4a* was 4.8 cM. An allele of *Pm4*, designated *Pm4d*, was derived from einkorn wheat (*T. monococcum* L.) and flanked by SSR markers *Xgwm526* and *Xbarc122* at genetic distances of 3.4 and 1.0 cM, respectively (Schmolke et al., 2012). Because of their less close association with the target gene, these markers may not effectively detect *Pm4b.* Recently, Li et al. (2017) reported a new allele in the *Pm4* locus in the common wheat line D29, designated *Pm4e*, which was flanked by SSR markers *Xgdm93* and *Xhbg327* and co-segregated with STS markers *Xsts_bcd1231* and *TaAetPR5*. However, the relationship between these markers and *Pm4b* was not clear.

Common wheat is a hexaploid species (2*n* = 6*x* = 42; AABBDD genomes) with a large sized genome (∼17 Gbp) and ∼90% repetitive sequences (Gupta et al., 2008; Shewry, 2009), so traditional classes of molecular markers, such as RFLP, RAPD (Fabritius et al., 1997), SSR (Duan et al., 2003; Cheema et al., 2008; Tsilo et al., 2009), amplified fragment length polymorphism (AFLP) (Cai et al., 2003; Asnaghi et al., 2004), as well as cleaved amplified polymorphic sequence (CAPS) for detecting single nucleotide polymorphism (SNP) (Lambreghts et al., 2009), cannot meet the demand for identifying closely linked markers due to inadequate density and high levels of duplication. Bulked segregant analysis-RNA-Seq (BSR-Seq) is a new genetic mapping strategy that combines the power of bulked segretant analysis (BSA) (Michelmore et al., 1991) and the ease of RNA-Seq technique. Using this strategy, Liu et al. (2012) mapped the genes in the population for which even no polymorphic markers were previously identified, resulting in cloning of *glossy3* (*gl3*) gene from maize (*Zea mays* L.). Trick et al. (2012) sequenced mRNA from the NILs spanning a ∼30 cM interval including the *GPC-B1* locus and two bulked samples that consisted of homozygous recombinant lines contrasting for their grain protein content (GPC) phenotypes. After discriminating for SNPs from the RNA-Seq data between the two NILs, they identified 39 new SNP markers, corresponding to 67% of the validated SNPs, mapped across a 12.2 cM interval including *GPC-B1*, and defined this gene to an interval containing 13–18 genes in the syntenic cereal genomes within a 0.4 cM interval of wheat. Ramirez-Gonzalez et al. (2015) combined BSA and the next generation sequencing technique to construct a high density genetic map of wheat and localized the stripe rust (caused by *Puccinia striiformis* West.) resistance gene *Yr15* to a 0.77 cM interval. In a recent study, *YrMM58* and *YrHY1* for resistance to stripe rust were mapped in the distal ∼16 Mb region on chromosome 2AS (Wang et al., 2017). These studies have demonstrated that BSR-Seq is effective to identify SNP markers for fine-mapping and even cloning target genes, especially in the genome regions with low polymorphism.

The objectives of this study were to characterize the resistance of *Pm4b* to different *Bgt* isolates that were collected from China and to develop closely linked markers that can be used to detect *Pm4b* using BSR-Seq technique and comparative genomics approach. Results of this study will be useful for marker-assisted breeding and pyramiding *Pm4b* with other resistance genes for the improvement of wheat against powdery mildew.

## Materials and Methods

### Plant Materials

In 2005, *Pm4b* was transferred from VPM1 [pedigree: *Aegilops ventricosa*/*T. turgidum* L. var. *carthlicum* (*T. persicum*)//3^∗^ *T. aestivum* cv. Marne] to the Chinese winter wheat cultivar Bainong 3217 resulting in the production of the *Pm4b* NIL VPM1/7^∗^Bainong 3217 F_4_ (Zhou et al., 2005). The susceptible recurrent parent Bainong 3217, with the pedigree of [(Funo × Neixiang 5) F_1_ × Xiannong 39] F_2_ × (Xinong 64(4)43 line 2 × Yanda 24) F_1_, was widely grown in the Huang and Huai Rivers Valley Winter Wheat Zone (Huang et al., 1982). In 2013, line VPM1/7^∗^Bainong 3217 F_4_ was crossed with Bainong 3217 to produce F_1_, F_2_, and F_2:3_ populations to be used in the genetic analysis and molecular mapping of *Pm4b*. A set of 23 differential wheat cultivars or lines that carry known powdery mildew resistance genes was used to differentiate the *Bgt* isolates. Twenty-seven wheat entries carrying known powdery mildew resistance genes and 46 wheat cultivars were used to validate the *Pm4b*-lined markers developed in the present study. Winter wheat cultivars Zhongzuo 9504 was used as the susceptible control in the assessments of powdery mildew resistance.

### Evaluation of Resistance to Powdery Mildew at the Seedling Stage

Genetic analysis and molecular mapping of the target resistance gene were conducted using the F_1_, F_2_, and F_2:3_ populations derived from the cross VPM1/7^∗^Bainong 3217 F_4_ × Bainong 3217. The parents and the mapping populations were grown in plastic trays with 5 × 10 wells (5 cm × 5 cm × 5 cm in dimension). At least 15 plants were tested for each F_2:3_ family. The conidiospores of *Bgt* isolate E20 freshly increased on the susceptible cultivar Zhongzuo 9504 were dusted on the tested seedlings at one-leaf stage. The inoculated plants were grown in a greenhouse at 20°C/14°C (day/night) with a photoperiod of 16 h light/8 h dark. Fifteen days after inoculation when the susceptible control Zhongzuo 9504 plants were heavily diseased, infection type (IT) of each plant was visually rated on a 0–4 scale as described by Liu et al. (1999). Plants were classified into the resistant group when the ITs were 0–2 or the susceptible group when the ITs were 3–4. Forty-six *Bgt* isolates were used to determine the effectiveness of *Pm4b* against powdery mildew using the same method as described above. These isolates were purified at least three times by the single colony method after they were collected from different wheat fields located in the provinces of Hebei, Shandong, Henan, Shanxi, Beijing, Jiangsu, Yunnan, and Guizhou in China.

### Genotyping of F_2:3_ Lines Using BSR-Seq Analysis

The BSR-Seq approach was performed on selected F_2:3_ families from the mapping population of VPM1/7^∗^Bainong 3217 F_4_ × Bainong 3217 cross. The representative plants from each F_2:3_ family with known *Bgt*-resistant/susceptible phenotypes were grown in a *Bgt*-free growth chamber. The phenotypically contrasting bulks of leaf samples were created by pooling equal size of the primary leaf from each representative plant two-leaf-old of 50 homozygous resistant and 50 homozygous susceptible F_2:3_ families. Total RNA of the two bulks of leaf samples was separately extracted using the Illumina TruSeq RNA Sample Prep Kit (Illumina, Inc., San Diego, CA, United States) to be used in RNA-Seq analysis using the platform of Illumina HiSeq 4000 (Beijing Southern Genome Research Technology Co., Ltd., Beijing, China). The raw sequencing reads generated were quality controlled using software Trimmomatic v0.36 (Bolger et al., 2014) with the default parameters. Using software STAR v2.5.1b (Dobin et al., 2013), the clean reads were aligned to the Chinese Spring whole genome assembly sequences (IWGSC WGS v1, NRGene DeNovoMAGIC, Seq Repository of Wheat Portal on URGI, INRA, France^2^ with the mismatch rate of less than 5%. The uniquely mapped read pairs were used in further analysis. The read alignments were masked for PCR duplications and split for reads spanning introns before they were used to call SNPs and InDels using module “HaplotypeCaller” software GATK v3.6 (McKenna et al., 2010). The resulting SNPs and InDels with sequencing depth less than 6 were discarded, and the remaining ones were applied to BSA. Only variants with allele frequency difference (AFD) > 0.6 and *P*-value of Fisher’s exact test on read count data < 1*e*-8 were classified as resistance-associated variants and used as templates for marker development.

### Development of SNP and SSR Markers

The SNPs associated with the powdery mildew resistance identified by BSR-Seq analysis were selected for marker development. The flanking sequences approximately 3 kb of the candidate SNPs were used as templates for designing PCR primers using the web-based program available at GSP website^3^. The closest SNP markers were used as queries for BLAST against the Chinese Spring whole genome assembly sequences (IWGSC WGS v1, NRGene DeNovoMAGIC, Seq Repository of Wheat Portal on URGI, INRA, France). The genomic sequences located downstream of the *Pm4b*-linked SNP marker (*2AL71*) developed in this study were used as templates to design SSR primers using batchprimer3^4^. Polymorphic SSR markers between the parents and the contrasting DNA bulks were used to construct the genetic linkage map of *Pm4b*.

### DNA Amplification and Electrophoresis

Genomic DNA was extracted from the fresh leaf tissues from each family of the F_2:3_ mapping population, following the cetyltrimethylammonium ammonium bromide (CTAB) method (Saghai-Maroof et al., 1984). The resistant and susceptible DNA pools were created by separately bulking equal amount of DNA from 16 resistant and 16 susceptible F_2:3_ families for detecting the polymorphism of SNPs and SSR markers. PCR was performed in a Biometra T3000 Thermocycler (ABI, New York, NY, United States). A reaction mixture (10 μL) consisted of 50–100 ng of template DNA, 0.4 μM each of the forward and reverse primers, 1 U of *Taq* polymerase, 0.4 mM dNTPs, and 2 μL 10× buffer with 20 mM Mg^2+^. Amplification of DNA was programmed at 94°C for 4 min; 35 cycles of 94°C for 45 s, 52–60°C for 45 s, and 72°C for 1 min. The reaction was terminated after an extension at 72°C for 10 min. The resulting PCR products were mixed with 2 μL loading buffer (98% formamide, 10 mM EDTA, 0.25% bromophenol blue, and 0.25% xylene cyanol) prior to separation on 1–2% agarose gel or 8% non-denaturing polyacrylamide gel (Acr:Bis = 19:1 or 39:1).

### Physical Mapping and Comparative Genomics Analysis

The sequences of SSR markers *Xics13* and *Xics43* that flanked *Pm4b* were used to search against the genomic regions of the Chinese Spring whole genome assembly sequences (IWGSC WGS v1, NRGene DeNovoMAGIC, Seq Repository of Wheat Portal on URGI, INRA, France) and the Chinese Spring cDNA sequence information was used to obtain the genes that were included in the interval of the two *Pm4b*-flanking markers. Then, these genes were annotated by the online programs EnsemblPlants^5^ and NCBI^6^. These online databases provide the annotation information for the genes of *T. aestivum* and the homologous genes of *Brachypodium distachyon* (L.), rice (*Oryza sativa* L.), and sorghum (*Sorghum bicolor* L.).

### Statistical Analysis and Linkage Map Construction

The Chi-squared test (χ^2^) for the goodness of fit was performed to determine the deviations of observed data from the expected segregation ratios using SAS 8.0 statistical analysis package (SAS Institute, Cary, NC, United States). Linkage between markers and the target resistance gene was established with the software Mapmaker/Exp Version 3.0b (Lincoln et al., 1993). Genetic distances were determined using the Kosambi function. The logarithm of the odds ratio (LOD) threshold score was set at 3.0 and the maximum distance allowed between markers was set at 50.0 cM.

## Results

### Characterization of *Pm4b* Resistance to Different *Bgt* Isolates

Forty-six *Bgt* isolates were used to examine the virulence spectrum against *Pm4b* in line VPM1/7^∗^Bainong 3217 F_4_. These isolates produced different ITs on the differential wheat cultivars or lines with known powdery mildew resistance genes (Supplementary Table S1). Line VPM1/7^∗^Bainong 3217 F_4_ was resistant to 52.2% of the isolates tested, while Khapli/8^∗^Cc carrying *Pm4a* was resistant to 39.1% of them. Line VPM1/7^∗^Bainong 3217 F_4_ was resistant to 72.7% of isolates that were collected from Hebei province, and it was effective against half of the isolates from Henan, and Shandong provinces. Bainong 3217 was as susceptible as the control Zhongzuo 9504 to all the *Bgt* isolates tested.

### Inheritance of Resistance to Powdery Mildew in Line VPM1/7^∗^Bainong 3217 F_4_

The pair of NILs VPM1/7^∗^Bainong 3217 F_4_ and Bainong 3217 differed in their reactions to *Bgt* isolate E20 (**Table 1**). Line VPM1/7^∗^Bainong 3217 F_4_ was highly resistant with an IT of 0, while the recurrent parent Bainong 3217 was highly susceptible with an IT of 4. The F_1_ plants derived from the cross between VPM1/7^∗^Bainong 3217 F_4_ and Bainong 3217 produced ITs that were similar to the resistant line VPM1/7^∗^Bainong 3217 F_4_. The F_2_ plants exhibited a 3:1 segregation ratio for the resistant and susceptible plants and the F_2:3_ population showed a 1:2:1 segregating ratio for the homozygously resistant, heterozygous, and homozygously susceptible families (**Table 1**). All these results demonstrate that the resistance to *Bgt* isolate E20 in line VPM1/7^∗^Bainong 3217 F_4_ inherits in a mode of single dominant gene.

**Table 1:** Classification of responses to the *Blumeria graminis* f. sp. *tritici* isolate E20 for the F_1_, F_2_, and F_2:3_ progenies derived from VPM1/7^∗^Bainong 3217 F_4_ × Bainong 3217 cross.

Parent/cross	Generation	Total number of plants/families	Observed ratio			Expected ratio	χ^2^	*P*-value


			HR	Seg	HS			
VPM1/7^∗^Bainong 3217 F_4_	P_R_	25	20		0			
Bainong 3217	P_S_	25	0		20			
VPM1/7^∗^Bainong 3217 F_4_ × Bainong 3217	F_1_	25	20		0			
VPM1/7^∗^Bainong 3217 F_4_ × Bainong 3217	F_2_	337	248		89	3:1	0.286	0.593
VPM1/7^∗^Bainong 3217 F_4_ × Bainong 3217	F_2:3_	288	72	149	67	1:2:1	0.521	0.771


*P_*R*_, resistant parent; P_*S*_, susceptible parent; HR, homozygous resistant, Seg, segregating, (heterozygous resistant); HS, (homozygous susceptible).*

### RNA-Seq Analysis of the Bulked RNA Pools with Distinct Reactions to Powdery Mildew

**FIGURE 1:**
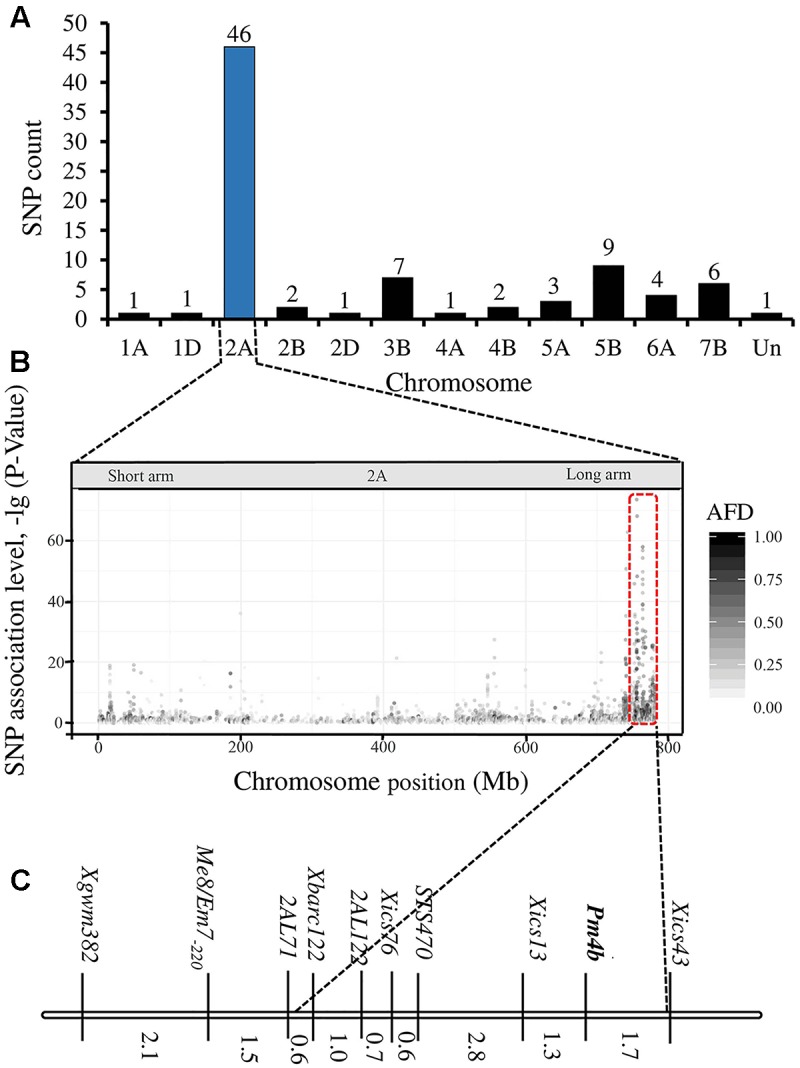
Overview of analyses. Number of polymorphic SNPs distributed on the different chromosomes **(A)** (Un means the unmapped SNPs), distribution of single nucleotide variation on wheat chromosomes **(B)**, and linkage map of *Pm4b* from this study **(C)**.

The RNA-Seq analysis generated 22,700,130 and 25,870,804 raw read pairs for the powdery mildew resistant and susceptible bulks, respectively. After quality control process, the resistant bulk remained 20,745,939 high-quality read pairs and 18,217,476 (82.8%) of them were uniquely mapped to the Chinese Spring wheat whole genome draft assembly. There were 25,867,480 high-quality and 20,741,967 (80.2%) uniquely mapped reads for the susceptible bulk. Variant calling identified 283,866 raw SNPs and InDels between the two bulks, and 101,835 of them had a depth > 6. The SNPs with high association level focused on chromosome 2AL (**Figures 1A,B**). Results of BSA revealed that 84 variants were potentially associated with the target powdery mildew resistance gene. These SNPs mainly distributed on chromosome 2AL, which indicates that the resistance gene is located on this chromosome arm. Forty-six SNPs were enriched in an about 25 Mb region in the distal part of chromosome 2AL (Supplementary Table S2).

### Polymorphic Analysis of Specific Primers Designed Based on the SNP Calling

**FIGURE 2:**
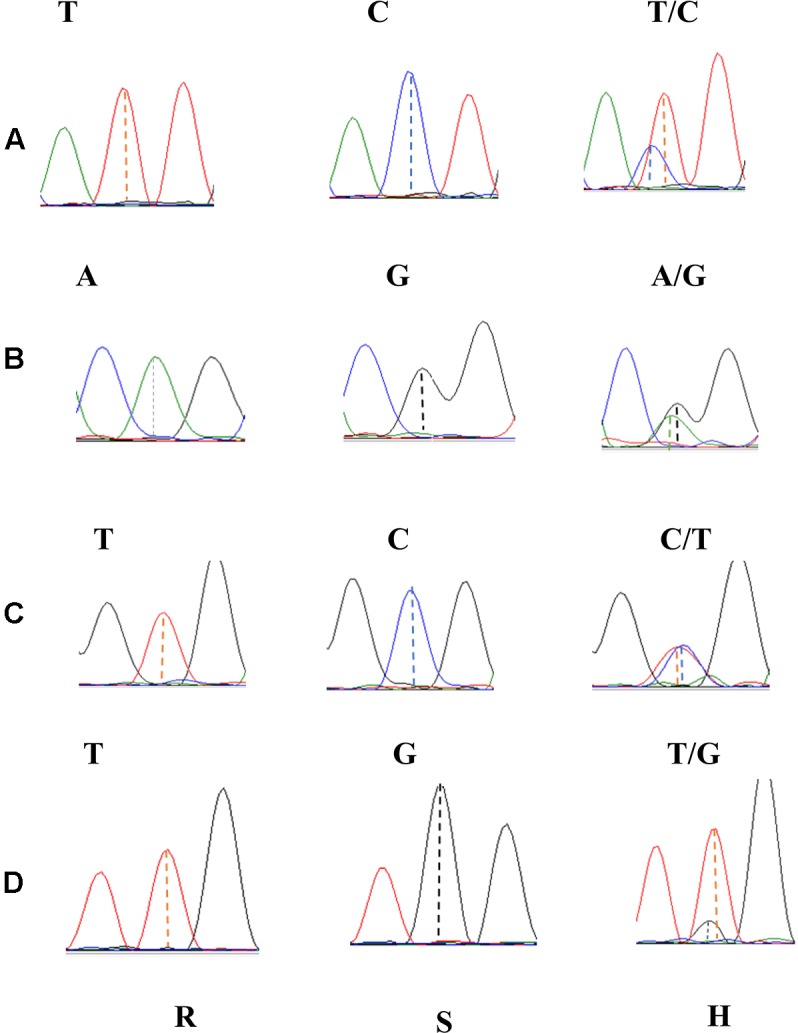
Sanger sequencing profiles of SNP markers *2AL43* **(A)**, *2AL71* **(B)**, *2AL83* **(C)**, and *2AL122* **(D)** in homozygous resistant (R), homozygous susceptible (S), and heterozygous F_2:3_ families (H).

Using the 46 SNP-containing sequences Blast analysis resulted in 14 homologous scaffolds from the Chinese Spring genomic sequence. These scaffolds were used as templates for designing 53 pairs of SNP primers on the GSP website, and 12 of them produced specific primers (Supplementary Table S3). These primer pairs amplified 700–900 bp nucleic acid sequences containing the SNP variants in line VPM1/7^∗^Bainong 3217 F_4_, Bainong 3217 and the two contrasting bulked F_2:3_ families. Sequence analysis of the resulting amplicons confirmed the consistency of polymorphisms for the SNP variants between the parents and the two contrasting DNA bulks. The SNP markers *2AL43*, *2AL83*, *2AL71*, and *2AL122* were polymorphic between the parents and two contrasting bulked F_2:3_ families (**Figure 2**), indicating that they were possibly linked to *Pm4b*.

### Development of SSR Markers and Construction of Genetic Linkage Map

**FIGURE 3:**
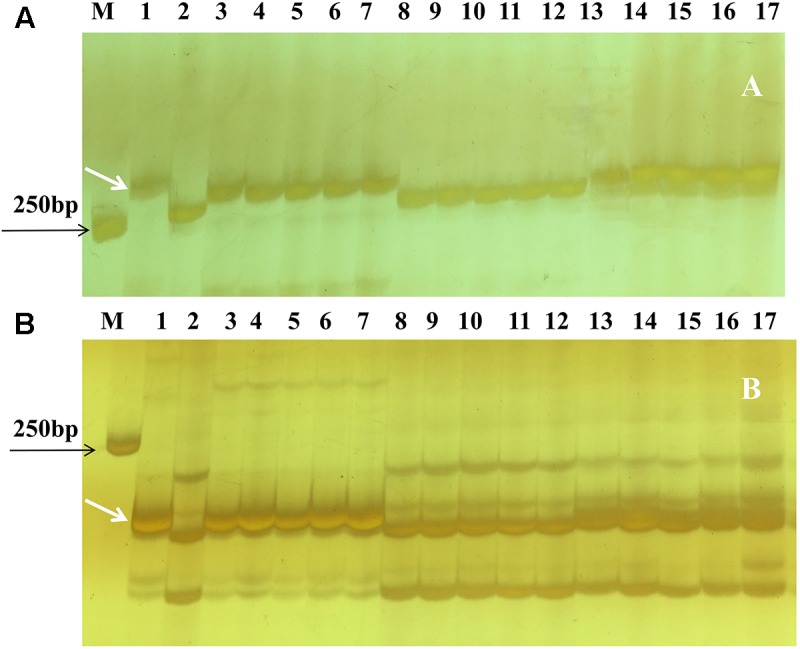
Amplification patterns of *Pm4b*-linked polymorphic SSR markers *Xics13* **(A)** and *Xics43* **(B)** in the parents and selected F_2:3_ Families of VPM1/7^∗^Bainong 3217 F_4_ × Bainong 3217 in 8% silver-stained non-denaturing polyacrylamide gels. Lane M, DL2000; lane 1: VPM1/7^∗^Bainong 3217 F_4_; lane 2: Bainong 3217; lanes 3–7: homozygous resistant F_2:3_ families; lanes 8–12, homozygous susceptible F_2:3_ families; and lanes 13–17: heterozygous F_2:3_ families. White arrows indicate the polymorphic bands specific for *Pm4b*.

Based on the linkage analysis, the SNP markers *2AL43*, *2AL83*, *2AL71*, and *2AL122* were located on the same side of *Pm4b* (**Table 2**), and the corresponding physical locations in the Chinese Spring reference sequences were 755,793,708, 770,411,718, 772,336,969, and 774,673,558 on the distal end of chromosome arm 2AL, respectively. Based on the information on the sites of these SNPs, *Pm4b* was located at a position between marker *2AL122* and the end of chromosome 2AL. Then, the genome sequence from the corresponding location of *2AL71* to the end of chromosome arm 2AL was used as template to design SSR primers (Supplementary Table S4). Among the 98 pairs of SSR primers designed, twelve were polymorphic between the two parents. Markers *Xics13*, *Xics43*, and *Xics76* were polymorphic between the contrasting DNA bulks, indicating their possible linkage to *Pm4b*. Based on their amplification patterns, *Xics13* and *Xics43* were co-dominant. *Xics13* amplified 264 bp and 252 bp bands, and *Xics43* produced 201 bp and 217 bp bands in the resistant and susceptible individuals of the mapping population, respectively (**Figure 3**). The dominant marker *Xics76* produced a 167 bp fragment from the resistant individuals and null from the susceptible individuals. In addition, the polymorphism of the published *Pm4*-linked markers was examined using the F_2:3_ mapping population. Four markers *STS*_470_, *Xbarc122*, *Me8*/*Em7*_-220_, and *Xgwm382* were polymorphic between the parents and the bulks (**Table 3**). *Xgwm356* specific for *Pm4a* was not incorporated into the new genetic linkage map of *Pm4b* because it was not polymorphic between the bulks. The other molecular markers that were previously linked to *Pm4b*, *Pm4c*, *Pm4d*, and *Pm4e* were not polymorphic between neither the parents nor the bulks of the current mapping population (**Table 3**). Therefore, the newly developed polymorphic SNP markers *2AL71* and *2AL122* and SSR markers *Xics13*, *Xics43*, and *Xics76*, together with the four polymorphic *Pm4-*linked markers, were used to construct the genetic linkage map after genotying the F_2:3_ mapping population (**Figure 1C**). In this linkage map, the SNP markers *2AL122* and *2AL71* were closer to *Pm4b* than the previously identified markers, except for *STS*_470_. The newly designed SSR markers *Xics76*, *Xics13*, and *Xics43* were located on the distal side to the SNP markers and closer to the target gene. *Pm4b* was flanked by markers *Xics13* and *Xics43* with genetic distances of 1.3 and 1.7 cM, respectively.

**Table 2:** Graphical genotypes of the SNP markers for the 16 F_2:3_ individuals used to develop the markers closely linked to *Pm4b.*

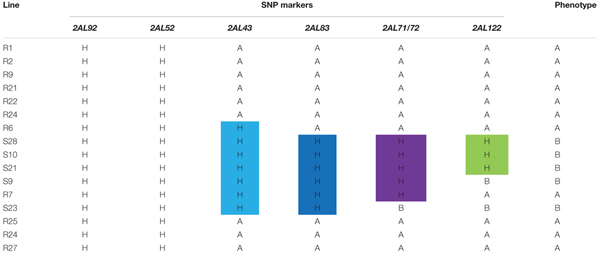

*A, resistant phenotype or genotype; B, susceptible phenotype or genotype; H, heterozygous phenotype or genotype.*

**Table 3:** Analysis of polymorphism in the mapping population of VPM1/7^∗^Bainong 3217 F_4_ × Bainong 3217 with the markers located on *Pm4* locus on chromosome 2AL.

Marker	*Pm* gene	Polymorphic between		Linkage to *Pm4b*	Reference
				
		Parents	Bulks		
*Xgwm356*	*Pm4a*	+	-	N/A	Ma et al., 2004; Hao et al., 2008
*STS*_470_	*Pm4a*	+	+	+	Ma et al., 2004
*Me12*	*Pm4b*	-	-	N/A	Yi et al., 2008
*STS*_-241_	*Pm4b*	-	-	N/A	Yi et al., 2008
*Me8/Em7_-220_*	*Pm4b*	+	+	+	Yi et al., 2008
*Xgwm382*	*Pm4b*	+	+	+	Yi et al., 2008
*Xbarc122*	*Pm4c*	+	+	+	Hao et al., 2008; Schmolke et al., 2012
*Xbarc76*	*Pm4c*	-	-	N/A	Hao et al., 2008
*Xgwm526*	*Pm4d*	-	-	N/A	Schmolke et al., 2012
*Xgdm93*	*Pm4e*	-	-	N/A	Li et al., 2017
*Xhbg327*	*Pm4e*	-	-	N/A	Li et al., 2017
*Xsts_bcd1231*	*Pm4e*	-	-	N/A	Li et al., 2017
*TaAetPR5*	*Pm4e*	-	-	N/A	Li et al., 2017


*+, polymorphic or linked; –, non-polymorphic or unlinked; N/A, not available.*

### Validation of Markers *Xics13* and *Xics43*

To validate the *Pm4b*-flanking markers, *Xics13* and *Xics43* were used to amplify 27 wheat differential cultivars or lines that carry known *Pm* genes. The target bands were amplified only from line VPM1/7^∗^Bainong 3217 F_4_, but not from the other 26 wheat accessions (**Table 4**). Meanwhile, markers *Xics13* and *Xics43* were used to genotype *Pm4b* in a panel of 46 wheat cultivars. Lankaoaizao 8 showed the same banding patterns as line VPM1/7^∗^Bainong 3217 F_4_, indicating that it may carry *Pm4b* (**Table 5**). However, the other 45 cultivars showed the same banding patterns as Bainong 3217, indicating the absence of *Pm4b*. This indicates that *Xics13* and *Xics43* are diagnostic molecular markers linked to *Pm4b*, which can be used in the marker-assisted selection (MAS) program for detecting and pyramiding *Pm4b* with other genes in the breeding program.

**Table 4:** Validation of *Pm4b*-linked SSR marker *Xics13* and *Xics43* on 27 Chinese wheat cultivars or lines with known powdery mildew resistance genes.

Cultivar	Gene	*Xics13*	*Xics43*
Xiaobaidongmai	*Mlxbd*	-	-
Hongquanmang	*PmH*	-	-
Liangxing 66	*PmLX66*	-	-
Uika/8^∗^Cc	*Pm1c*	-	-
Zhongmai 155	*Pm2*	-	-
Sonom/8^∗^Cc	*Pm3c*	-	-
7P580	*Pm3e*	-	-
Courtot	*Pm3g*	-	-
Khapli/8^∗^Cc	*Pm4a*	-	-
VPM1/7^∗^Bainong 3217	*Pm4b*	+	+
Line 81-7241	*Pm4c*	-	-
CII4125	*Pm5a*	-	-
Fuzhuang 30	*Pm5e*	-	-
Coker 747	*Pm6*	-	-
CI 141879	*Pm7*	-	-
PI361879	*Pm8*	-	-
CI 14119	*Pm12*	-	-
Bainong 3217	*Pm13*	-	-
96-283	*Pm16*	-	-
96-287	*Pm20*	-	-
Jinhe 9127	*Pm21*	-	-
Chiyacao	*Pm24*	-	-
MG29896	*Pm36*	-	-
2636-24R	*Pm43*	-	-
Tabasco	*Pm46*	-	-
Hongyanglazi	*Pm47*	-	-
Liangxing 99	*Pm52*	-	-


*+, amplified marker site produced the target band; -, amplified marker site did not produced the target band.*

**Table 5:** Validation of SSR marker *Xics13* and *Xics43* on 46 Chinese wheat cultivars in MAS.

Cultivar	Origin of province	*Xics13*	*Xics43*
Annong 1008	Anhui	-	-
Wanmai 52	Anhui	-	-
Jingdong 8	Beijing	-	-
Lunxuan 136	Beijing	-	-
Jinhe 9123	Hebei	-	-
Gaoyou 503	Hebei	-	-
Gaocheng 8901	Hebei	-	-
Han 6172	Hebei	-	-
Heng 4399	Hebei	-	-
Hengguan 35	Hebei	-	-
Shijiazhuang 8	Hebei	-	-
Xingmai 6	Hebei	-	-
Jinfeng 3	Henan	-	-
Kenong 199	Henan	-	-
Zhou 8425B	Henan	-	-
Bainong 160	Henan	-	-
Fanmai 5	Henan	-	-
Fanmai 8	Henan	-	-
Fengdecunmai1	Henan	-	-
Fengwu 981	Henan	-	-
Huapei 5	Henan	-	-
Huapei 8	Henan	-	-
Huaichuan 916	Henan	-	-
Kaimai 20	Henan	-	-
Lankaoaizao 8	Henan	+	+
Luomai 4	Henan	-	-
Pingan 6	Henan	-	-
Pingan 8	Henan	-	-
Yangmai 13	Jiangsu	-	-
Yangmai 16	Jiangsu	-	-
Jimai 26	Shandong	-	-
Jimai 38	Shandong	-	-
Jimai 20	Shandong	-	-
Jimai 21	Shandong	-	-
Jimai 22	Shandong	-	-
Jinan 17	Shandong	-	-
Liangxing 99	Shandong	-	-
Lumai 7	Shandong	-	-
Lumai 21	Shandong	-	-
Lumai 23	Shandong	-	-
Shangdong 20	Shandong	-	-
Taishang 23	Shandong	-	-
Weimai 8	Shandong	-	-
Yanfu 188	Shandong	-	-
Yannong 19	Shandong	-	-
Jimai 30	Shandong	-	-


*+, amplified marker site produced the target band; -, amplified marker site did not produced the target band.*

### Physical Mapping and Comparative Genomics Analysis

**FIGURE 4:**
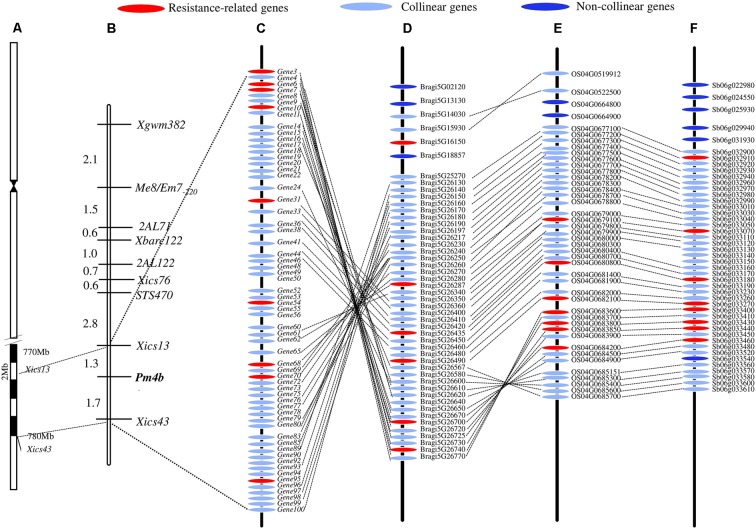
Schematic diagram of *Pm4b* physical interval on the chromosome position **(A)**, genetic linkage map **(B)**, and synteny genes among *T. aestivum* cv. Chinese Spring **(C)** and *Brachypodium distachyon* **(D)**, rice **(E)**, and sorghum **(F)** in the *Pm4b* corresponding genomic interval. *Pm4b* is located in an about 6.7 Mb physical interval on the end of chromosome 2AL, and the collinear genes and resistance-related genes among *T. aestivum* cv. Chinese Spring, *B. distachyon*, rice and sorghum in the *Pm4b* corresponding genomic interval are shown.

The DNA sequences associated with the *Pm4b*-flanking markers *Xics13* and *Xics43* were positioned on the Chinese Spring reference genome sequence. The existing cDNA sequence information of the Chinese Spring database was used to enucleate the genes in the genomic regions between the two *Pm4b*-flanking markers. This region spanned a physical interval of about 6.7 Mb (773,680,429–780,760,825) on the Chinese Spring chromosome 2AL and contained 101 predicted genes. This region displayed a collinear relationship with *B. distachyon* in a 0.99 Mb (26,657,763–27,645,510) genomic region containing 49 predicted genes on chromosome 5, a 1.20 Mb (33,028,549–34,234,325) genomic region containing 47 predicted genes on rice chromosome 4, and a 0.61 Mb (60,898,697–61,519,884) genomic region containing 36 predicted genes on sorghum chromosome 6 (**Figure 4**). Seven transcripts, which encode for disease resistance-associated proteins, such as C2 domain, peroxidase activity protein, protein kinases of PKc_like super family, Mlo family protein, and catalytic domain of the serine/threonine kinases (STKc_IRAK like super family), were identified in this collinear genomic regions (**Table 6**).

**Table 6:** Disease resistance genes identified in the *Pm4b* collinear genomic regions in *Triticum aestivum* cv. Chinese Spring, *Brachypodium distachyon*, rice, and sorghum.

Code	Chinese Spring	*B. distachyon*	Rice	Sorghum	Annotation
1	*CS_gene6*	*BRADI5G26730*	*Sb06g033430*	*OS04G0683800*	Most C2 domain protein
2	*CS_gene7*	*BRADI5G26725*	*Sb06g033440*	*OS04G0683850*	peroxidase activity
3	*CS_gene10*	*BRADI5G26700*	*Sb06g033460*	*OS04G0684200*	PKc_like super family, protein kinases, catalytic domain
4	*CS_gene31*	*BRADI5G26490*	*Sb06g033270*	*OS04G0682100*	C2 domain
5	*CS_gene54*	*BRADI5G26435*	*Sb06g033180*	*OS04G0680800*	Mlo family
6	*CS_gene68*	*BRADI5G26287*	*Sb06g033070*	*OS04G0679100*	STKc_IRAK, catalytic domain of the serine/threonine kinases
7	*CS_gene95*	*BRADI5G26770*	*Sb06g033400*	*OS04G0683600*	PKc_like super family, protein kinases, catalytic domain


## Discussion

*Pm4b* was effective in certain areas of wheat producing fields in China. Results of BSR-Seq analysis demonstrated that *Pm4b* was located on the distal end of chromosome arm 2AL, which is consistent with its localization in previous study (Bariana and McIntosh, 1993). Three newly developed SSR markers, *Xics13*, *Xics43*, and *Xics76*, were incorporated into the genetic linkage map of *Pm4b*. These markers were able to produce the diagnostic banding patterns for clearly distinguishing *Pm4b* from other known *Pm* genes. The identification of these markers will facilitate molecular detection of *Pm4b* in MAS programs or pyramiding it with other effective genes for providing a broader spectrum of resistance against powdery mildew.

*Pm4b* was derived from a *T. persicum* chromosomal segment that was introgressed onto wheat chromosome 2AL (Bariana and McIntosh, 1993). The lack of homeologous chromosome pairing may prevent the recombination between this *T. persicum* chromosomal segment and the corresponding wheat chromosome. Moreover, the poor abundance of markers applied in previous works impeded the identification of molecular markers closely linked to *Pm4b* (Hao et al., 2008; Yi et al., 2008). Based on the linkage analysis in the current study, all the previously identified gene-linked markers were located on the proximal side of *Pm4b* using the mapping population in this study (**Figure 1C**).

To develop more gene-linked markers, the BSR-Seq technique was applied with the mapping population derived from the *Pm4b* NILs. This technique resulted in the identification of four polymorphic SNP markers, which were anchored in the distal region of chromosome 2AL of the Chinese Spring wheat genome sequence. An interval (∼20 Mb) of the genomic sequence that flanked these SNP markers was used to develop SSR markers associated with the target gene. Among the 98 pairs of SSR primers designed from the sequence of this interval, three polymorphic markers were detected in the F_2:3_ mapping population, which were incorporated in the genetic linkage map of *Pm4b*. The transferability of SSRs between common wheat and the relative species varied (Kuleung et al., 2004). The low efficiency of polymorphic markers designed based on the Chinese Spring genome sequence may attribute to the origin of *Pm4b* from *T. persicum*. The emergence of new strategies provides a great potential to identify candidate genes of wheat. In the study of cloning *Pm21* gene that was transferred from chromosome 6VS of *Haynaldia villosa* L. to wheat chromosome 6AL, Xing et al. (unpublished) combined the strategies of development of cytogenetic stocks, mutagenesis, RenSeq and PacBio and identified an NBS-LRR type gene NLR1-V from the *Pm21* locus. He et al. (2017) identified *Pm21* as a typical coiled-coil-nucleotide-binding site, leucine-rich repeat (CC-NBS-LRR) gene by an integrated strategy of resistance gene analog (RGA)-based cloning via comparative genomics, physical and genetic mapping. Based on the flow sorting and sequencing of mutant chromosomes technique, Sánchez-Martín et al. (2016) developed the MutChromSeq technique to recognize induced mutations and isolated *Pm2* gene that was derived from *Ae. tauschii* Coss.

RNA-Seq is a way to look for the alien pieces with *de novo* assembly of the transcriptome data (Liu et al., 2012). For the species without the reference genome sequence, however, it may increase the difficulty to obtain effective Single Nucleotide Variants (SNVs), which prevents the identification of enough variants associated with the traits of interest. In such cases, the genome sequences of the homoeologous species are often used as the reference genomes. The challenges in *de novo* assembly of pooled RNA-Seq data in a huge genome of a hexaploid species such as wheat using only the mapped reads into high-quality and full-length transcripts may hinder the finding of introduced genes from other related species. Also, it may miss the expressed sequences in the gaps of the Chinese Spring reference genome sequences, the highly homologous and homoeologous sequences, the sequences dislike the Chinese Spring reference genome sequences, and the unique sequences from the related species. The technique of BSR-Seq often cannot obtain the induced expression information of disease-resistance genes taking into consideration that the RNA samples are extracted from leaves uninoculated with any *Bgt* isolates. Moreover, the effective use of this methodology is largely associated with the sequencing depth. The limited sequencing depth may be inadequate for calling reliable variants from low expressed genes for the purpose of association analysis. Sequencing in higher depth and longer length, improving the BSR-Seq in *de novo* assembly of low expressed genes, and optimizing algorithms of variant calling and allele frequency estimations in pooled RNA-Seq samples would be helpful to solve this problem.

Physical mapping of the *Xics13* and *Xics43* markers that were linked to *Pm4b* to the Chinese Spring reference genome sequence enabled the identification of candidate genes for disease resistance, for example, C2 domain, peroxidase activity protein, protein kinases of PKc_like super family, Mlo family protein, and catalytic domain of the serine/threonine kinases (STKc_IRAK like super family) (**Table 5**). Up to now, four wheat powdery mildew resistance genes, *Pm3b*, *Pm21*, *Pm2*, and *Pm60*, have been cloned in wheat. *Pm3b* is a member of the CC-NBS-LRR type of disease resistance genes (Yahiaoui et al., 2004). *Stpk-V*, a serine/threonine protein kinase gene, was shown to be a member of *Pm21* (Cao et al., 2011). In the most recent studies, *Pm21* proved to be the CC-NBS-LRR gene *NLR1-V* (He et al., 2017; Xing et al., unpublished). *Pm2* was also identified as a CC-NB-ARC-LRR resistance gene (Sánchez-Martín et al., 2016). *Pm60*, originating from *T. urartu* Thumanjan ex Gandilyan, is also a NB-LRR gene (Zou et al., 2017). The wild-type *Mlo* gene is a negative regulator of resistance to powdery mildew in barley (*Hordeum vulgare* L.) (Büschges et al., 1997). Currently, over 100 *R* genes have been cloned from various species, and the NBS-LRR proteins are the most abundant class of disease resistance genes in plants (Yang et al., 2013). Based on the features of their N-terminal structures, this protein family includes two major subfamilies: the Toll-interleukin (TIR-NBS-LRR) subfamily and the coiled-coil (CC-NBS-LRR) subfamily (Krattinger and Keller, 2016). In the present study, no NBS-LRR type of resistance gene was predicted in the target genomic region. There is a need to fine map *Pm4b* to narrow the genomic region that ensures the precisely identification of the candidate gene of *Pm4b*.

Zeng et al. (2014) reported that the mean frequency of virulent isolates on *Pm4b* was 42.5% out of 1082 *Bgt* isolates from the major wheat-growing regions of China, with the lowest virulence frequency of 16.7% for the isolates from the mid-Valley of the Yangtze River. Results of the present study also demonstrated the effectiveness of *Pm4b* in some provinces in northern part of China. Also, pyramiding multiple resistance genes is another effective means to improve disease resistance. Mwale et al. (2017) detected 24 cultivars that carried *Pm4b* gene among 60 wheat cultivars from China using the gene-linked molecular markers. Based on that study, the combination of genes *Pm2*+*Pm4b*+*Pm8* was possibly present in cultivars Xinxuan 2039, Lankao 008 and Zhengmai 366, and Yumai 368 may possess *Pm2*+*Pm4b*+*Pm6*. Using the gene-linked markers, Zhang et al. (2002) identified 11 wheat lines that pyramided *Pm4b*, *Pm13*, and *Pm21* genes. The lines with multiple genes provided better resistance to powdery mildew than the single gene. Line VPM1/7^∗^Bainong 3217 F_4_, which was developed using VPM1 as the donor parent and Bainong 3217 as the recurrent parent, has promising agronomic traits in addition to the resistance to powdery mildew (Zhou et al., 2005). This ensures its direct application in the breeding programs in China. The development of the breeder-friendly PCR markers can facilitate the effective identification of *Pm4b* in the breeding populations.

In summary, four SNP and three SSR markers were developed by means of the BSR-Seq technique, which mapped *Pm4b* gene in a 3.0 cM genetic interval corresponding to a 6.7 Mb genomic region. The putative genes in this interval were annotated by the web-based programs EnsemblPlants and NCBI. This interval had a good collinearity with certain genomic regions of *B. distachyon* (chromosome 5), rice (chromosome 4) and sorghum (chromosome 6). The collinear genomic region contained seven disease resistance genes. *Xics13* and *Xics43* can be used as the diagnostic molecular markers for identifying *Pm4b* during its marker-assisted selection.

## Author Contributions

HjL and PW conceived and designed the study. PW, JH, and DQ conducted the experiments. PW, JX, JL, ML, and ZL analyzed the data. HZ, LY, and HwL performed the phenotypic tests and other works involved in this study. PW and HjL wrote the manuscript with the contributions of YZ, ZL, and ZZ.

## Conflict of Interest Statement

The authors declare that the research was conducted in the absence of any commercial or financial relationships that could be construed as a potential conflict of interest.

## Footnotes

1 https://shigen.nig.ac.jp/wheat/komugi/genes/download.jsp

2 https://wheat-urgi.versailles.inra.fr/Seq-Repository/News

3 https://probes.pw.usda.gov/GSP/

4 https://wheat.pw.usda.gov/demos/BatchPrimer3/

5 http://plants.ensembl.org/index.html

6 https://www.ncbi.nlm.nih.gov/Structure/cdd/wrpsb.cgi
